# Leader-Following Consensus and Formation Control of VTOL-UAVs with Event-Triggered Communications †

**DOI:** 10.3390/s19245498

**Published:** 2019-12-12

**Authors:** J. Fermi Guerrero-Castellanos, Argel Vega-Alonzo, Sylvain Durand, Nicolas Marchand, Victor R. Gonzalez-Diaz, Josefina Castañeda-Camacho, W. Fermin Guerrero-Sánchez

**Affiliations:** 1 Facultad de Ciencias de la Electrónica, Benemérita Universidad Autónoma de Puebla (BUAP), Cuidad Universitaria, Puebla 72570, Mexico; argel.vegaalonzo@icloud.com (A.V.-A.); vicrodolfo.gonzalez@correo.buap.mx (V.R.G.-D.); josefina.castaneda@correo.buap.mx (J.C.-C.); willi@fcfm.buap.mx (W.F.G.-S.); 2 ICube Laboratory, CNRS, INSA Strasbourg, Strasbourg University, 67000 Strasbourg, France; sylvain@durandchamontin.fr; 3 GIPSA-Lab, Grenoble INP, CNRS, Université Grenoble Alpes, 38000 Grenoble, France; nicolas.marchand@gipsa-lab.fr

**Keywords:** event-triggered control, VTOL-UAVs, consensus and formation control, multi-agent systems, cyber-physical systems (CPS)

## Abstract

This article presents the design and implementation of an event-triggered control approach, applied to the leader-following consensus and formation of a group of autonomous micro-aircraft with capabilities of vertical take-off and landing (VTOL-UAVs). The control strategy is based on an inner–outer loop control approach. The inner control law stabilizes the attitude and position of one agent, whereas the outer control follows a virtual leader to achieve position consensus cooperatively through an event-triggered policy. The communication topology uses undirected and connected graphs. With such an event-triggered control, the closed-loop trajectories converge to a compact sphere, centered in the origin of the error space. Furthermore, the minimal inter-sampling time is proven to be below bounded avoiding the Zeno behavior. The formation problem addresses the group of agents to fly in a given shape configuration. The simulation and experimental results highlight the performance of the proposed control strategy.

## 1. Introduction

This section aims to introduce the reader to the context of cyber-physical systems and specifically the networks of Unmanned Aerial Vehicles (UAVs). Recent research and challenges about distributive control for consensus and formation control of multi-agent systems are also presented. In particular, much attention is payed regarding the event-triggered paradigm and its application to the collaborative tasks. The second part gives the scope of the paper and the main contributions.

### 1.1. Background and Context

Cyber-physical systems (CPSs) integrate computer-based functions, like computing and networking, with physical components. In these systems, software and hardware are deeply interconnected. Each section operates at different spatial and temporal scales with different and multiple behaviors, interacting with each other in many ways that change with context. CPSs cooperate, self-organize, act on their environment, etc., …, making autonomous decisions. The applications include transportation systems, automation, security, smart cities, medical monitoring, agriculture, military operations, process control, or robotics [3]. A special collection of CPSs is the cyber-physical vehicle systems (CPVSs), which comprise terrestrial, underwater, and flying vehicles [4,5,6,7]. Among CPVSs, unmanned flying multi-vehicles are of interest in the industrial and the academic field. A focus has notably been given to the problem of agreement (consensus) and the formation of micro aerial vehicles with capabilities of vertical take-off and landing, so-called VTOL-UAV [8,9,10]. In this case, cooperative distributed control strategies are exciting, and the robotic and control community have developed a set of charming policies and proposals for large-scale multi-agent issues. The primary motivation behind this effort is that a set of organized vehicles is likely able to outperform an individual or sparse systems operating separately [11,12,13,14].

It is worth noticing that the above mentioned collaborative control approaches consider the systems represented through a continuous-time model. It is also assumed that each VTOL-UAV, also called *agent* hereafter, broadcasts its state and continuously accesses the neighbors’ states. This scheme considers that if the algorithms are implemented over digital platforms, the signal’s discretization effectively approaches the continuous-time states, thanks to the excellent capabilities of modern embedded data converters. However, in practice, the continuous sharing between aerial vehicles is highly resource-consuming. Notably in terms of computing and energy cost. Therefore, it is essential to determine how regularly agents should share information to keep the closed-loop features for the continuous-time case [15]. Actually, a co-design framework is mandatory in CPS to obtain a fair trade-off between efficiency and performance. Typically, the requirements for the physical layer are designed without considering those of the digital layer. A physical system must, therefore, be optimized in relation to the computer resources and, complementary, the physical resources must be considered when designing the digital environment.

In networked control, like multi-agent systems, the power requirement is directly related to the sensor’s sampling rate, which generally sets the sampling period at which the embedded computer updates the control inputs and communicates with neighbors. Consequently, it becomes costly to execute communication tasks periodically at a high rate. The event-based paradigm emerges as an option to reduce the usage of communication bandwidth in the system [16]. Contrary to the periodic paradigm, a so-called event-triggered control [17,18,19,20,21,22] computes and updates the control signals only when a specific condition is satisfied, i.e., when an event occurs. In the framework of collaborative control of linear multi-agent systems (MASs), an event-triggered cooperative control allows sharing information with the agents, only if necessary. This is the reason for the event-triggered distributed techniques becoming a trending issue around the control theory community, reporting extraordinary results, see [23,24,25,26,27,28,29]. In the specific case of the distributed consensus problem of MASs modeled by single-integrator and double-integrator, one of the first works reported is [23], which proposes a mechanism for event-triggered communication to solve the consensus problem. However, in this approach the event function’s threshold is state-dependent, which means that continuous communication among neighbors is necessary to evaluate the event function. To overcome this issue, the authors in [24,30] propose a novel event-triggered mechanism, where the event function’s threshold is state-independent. The main advantage is that the event function’s threshold only depends on its state, and each agent broadcasts its state to neighbors only when an event takes place. As a result, continuous communication among neighbors is no longer required. This approach is extended to general linear MASs in [31,32]. Early works, such as [33,34] propose event-triggered leader-follower consensus of multi-agent systems for tracking and flocking purposes. However, in these approaches, the event function’s threshold is state-dependent or at least velocity dependent [35]. Several bibliographical surveys exist on event-triggered control algorithms for multi-agent consensus [36,37,38,39]. In the more recent and in-depth ones [38,39], the authors pay particular attention to the resulting characteristics of the algorithm execution, including network topology, number of triggers, event-triggered control mechanism and consequences of incomplete information.

Though the event-triggered technique shows benefits and has attracted tremendous attention from theoretical and practical perspectives, due to communication and computation reduction, none of the works previously mentioned have been validated experimentally. Furthermore, to the best of the authors’ knowledge, an event-triggered consensus approach has not been exploited in the framework of autonomous aerial vehicles (except in our preliminary work [1]).

### 1.2. Contribution of the Work

This work explores the inclusion of event-based control to recent VTOL-UAVs system control philosophies. For the sake of reference, the work in [1] describes the distributive event-triggered control employed to the problem of consensus of a collection of VTOL-UAVs in real-time experiments. However, the work only addresses consensus without a leader. Furthermore, the consensus convergence is uncharted in the mentioned work. On the other hand, the work in [2] reports a collaborative event-triggered control strategy applied to the problem of leader-following formation of a UAVs set. The aforementioned work presents a more rigorously convergence analysis but considers the vehicle evolution only in the plane carrying a suspended load. Moreover, the results are only from simulations.

For comparison purposes with [1] and [2], it is important to notice that the present work proposes and physically implements a control strategy for the leader-following consensus of multi under-actuated VTOL-UAVs. The control strategy is based on an inner–outer loop control approach. Firstly, an inner control allows stabilizing the attitude of the aircraft vehicle, using a nonlinear quaternion-based control. This inner control also takes into account the maximal actuator capabilities for each VTOL-UAV. Secondly, an outer loop handles the event-triggered communication and control position of the multi-vehicles system. The objective is following a virtual leader intending to achieve aggregation and formation. Our proposal is in the sense of [24,25]. In [24], the agents’ dynamics are described by first and second order integrators, whereas in [25] the agent is described by general linear dynamics. Both approaches consider only the consensus problem and verify the results using only numerical simulation.

The current work represents the communication topology with undirected and connected graphs. With such an event-triggered strategy, the closed-loop stability analysis guarantees practical convergence to the leader. In the first place, the numerical simulations for the formation of four VTOL-UAVs depict the proposed control effectiveness. Furthermore, a real-time implementation using three miniature VTOL-UAVs and a motion tracking system experimentally shows the performance of the proposed approach. Also, this work discusses the design and real-time implementation of a distributive event-triggered control. As it was mentioned, we aim to show to the reader how to combine and applied topics such as multi-UAVs, event-triggered control, and distributive control. This last is one of our main contributions.

The document is constituted as follows. Section 2 includes the mathematical preliminaries discussing graph theory and modeling of one VTOL-UAV. Section 3 details the design of the internal control for the attitude and position stabilization of each vehicle. In Section 4, the event-triggered distributed control is detailed, for both leader-following consensus and formation control of a group of VTOL-UAVs. Section 5 is devoted to simulation and experimental results, highlighting the effectiveness of the proposed algorithm. Finally, conclusions and future trajectories are discussed in Section 6.

## 2. Preliminaries

This section presents the notations and the mathematical background used in this paper. In Section 2.1, the notations and mathematical symbols used in the article are introduced. In Section 2.2, a graph theory outlook is presented, which provides means to examine how the structure of the underlying communication topology among the agents leads to the global behavior of the system. Event-triggered communication and some definitions are presented in Section 2.3. Finally, the dynamical model of the VTOL-UAVs, which will be considered as agents in the rest of the paper, is shown in Section 2.4.

### 2.1. Notation

In the following, $∥\cdot ∥$ denotes the Euclidian norm for vectors and the induced 2-norm for matrices, respectively. ℝ denotes the set of real numbers where ${\mathbb{R}}_{+}$ denotes positive reals. Given a Matrix $A\in {\mathbb{R}}^{n\times n},\lambda_{\min}\left(A\right),\lambda_{\max}\left(A\right),\lambda_{i}\left(A\right)$, denote the minimum, maximum and *i*th eigenvalue of *A*, respectively. The symbol ∧ denotes the “or” logical connective.

### 2.2. Graph Theory

Consider a graph G={V,E} consisting of a set of vertices (or nodes) V=1,…,N and edges E∈V×V. If there is an edge (i,j) between nodes *i* and *j*, with 1≤i≤N and 1≤j≤N, then *i* and *j* are called adjacent, i.e., E=(i,j)∈V×V:i,jadjacent. An entry of the adjacency matrix A is defined by $a_{ij}=1$ if *i* and *j* are adjacent and $a_{ij}=0$ otherwise. Note that the diagonal elements of the adjacency matrix are all zero for a graph without any loop (as in the present paper). G is called undirected if (i,j)∈E⇔(j,i)∈E. A path from *i* to *j* is a sequence of distinct nodes, starting from *i* and ending with *j*, such that each pair of consecutive nodes is adjacent. If there is a path from *i* to *j*, then *i* and *j* are called connected. If all pairs of nodes in G are connected, then G is called connected. The distance d(i,j) between two nodes is the number of edges of the shortest path from *i* to *j*. The diameter d of G is the maximum distance d(i,j) over all pairs of nodes. The degree (or valency) matrix D of G is a diagonal matrix whose diagonal elements $d_{i}$ are equal to the cardinality of node *i*’s neighbor set $N_{i}=\left\{j\in V:\left(i,j\right)\in E\right\}$. The Laplacian matrix L of G is defined as L=D−A. For undirected graphs, L is symmetric and positive semi-definite, i.e., $L=L^{T}\geq 0$. The row sums of L are zero. Thus, the vector of ones 1 is an eigenvector corresponding to eigenvalue $\lambda_{i}\left(G\right)=0$, i.e., L·1=0. For connected graphs, L has exactly one zero eigenvalue, and the eigenvalues can be listed in increasing order $0=\lambda_{1}\left(G\right)<\lambda_{2}\left(G\right)\leq \ldots \leq \lambda_{N}\left(G\right)$. The second eigenvalue $\lambda_{2}\left(G\right)$ is called the algebraic connectivity.

In a leader-following configuration, the leader is represented by an extra vertex 0 and information is exchanged between the leader and the following agents which are in its neighborhood. The leader is a *virtual* system in the present study. Then, such a configuration can be defined with a graph $\bar{G}$, which consists of graph G, vertex 0 and edges between the leader 0 and its neighbors. Furthermore, let $G=\mathrm{diag}(g_{1},\ldots ,g_{N})$ be the diagonal matrix of pinning gains, $g_{i}>0$, describing the connections between the leader and the follower nodes [14,40]. In this paper, if $g_{i}=1$ the node *i* is said to be pinned to the leader, i.e., the node *i* observes the leader and an edge (0,i) is said to exist.

**Lemma** **1.** *[41] The matrix H=L+G corresponding to a graph $\bar{G}$ has the following properties:*

*1.*  *The matrix H has nonnegative eigenvalues;*
*2.*  *The matrix H is positive definite if and only if the graph $\bar{G}$ is connected.*

### 2.3. Dynamic Systems and Event-Triggered Communication

A graph G (with a set of *N* vertices V=1,…,N) which vertices are dynamic systems, also called *agents* afterwards, is denoted (G,x), where $x={\left(\begin{array}{cccc} x_{1}^{T} & x_{2}^{T} & \ldots & x_{N}^{T} \end{array}\right)}^{T}$ is a global state vector which contains the dynamics of each agent in the general form:

(1) $${\dot{x}}_{i}=f\left(x_{i},u_{i}\right)$$

where $x_{i}\in X_{i}\subset {\mathbb{R}}^{n_{i}}$ is the state vector and $u_{i}\in U_{i}\subset {\mathbb{R}}^{p_{i}}$ the control input vector of agent *i*, with 1≤i≤N. The transmission of information between agents is event-triggered in the present proposal. This basically means the use of two functions:

- An *event function* $\epsilon_{i}:X_{i}\times X_{i}\to \mathbb{R}$ that pinpoints if agent *i* needs ($\epsilon_{i}\leq 0$) or not ($\epsilon_{i}>0$) to transmit its state to other agents *j*, with $j\in N_{i}$ where $N_{i}$ is the node *i*’s neighbor set. The event function $\epsilon_{i}\left(\cdot \right)$ for agent *i* depends on its current state $x_{i}$ and a memory $m_{i}$ of $x_{i}$ last time $\epsilon_{i}$ became negative.
- A *(static distributed) feedback function* $u_{i}$. The feedback function $u_{i}\left(\cdot \right)$ takes the current state $x_{i}$ as input and memories $m_{i}$ of $x_{i}$ and $m_{j}$ of $x_{j}$. Therefore, the control law for agent *i* varies with respect to (i) its current state value $x_{i}$, (ii) its state last time an event occurred $m_{i}$, and also (iii) the state of its neighbors last time an event occurred $m_{j}$. The term *static* means the state $x_{i}$ is measured and not estimated by another dynamical system (like an observer). The term *distributed* means the control law for one agent *i* is only related to the neighbor set $N_{i}$, which is itself a subset of the set for all nodes, i.e., $N_{i}\subset V$.

### 2.4. VTOL-UAV Mathematical Model

Firstly, assume that a VTOL-UAV can be modeled as a rigid body.

#### 2.4.1. Attitude Representation

**Figure 1:**
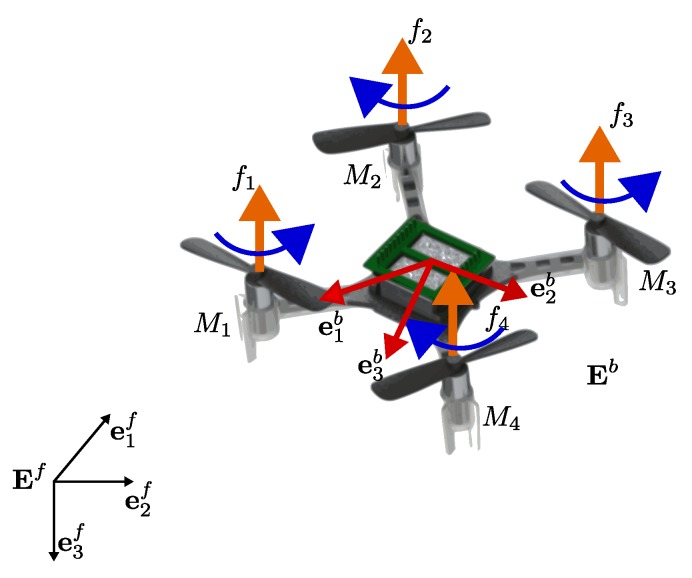
Body-fixed and inertial reference frames for a VTOL-UAV.

Consider two orthogonal right-handed coordinate frames: (i) the body coordinate frame $E^{i}=\left\{\begin{array}{ccc} e_{1}^{i} & e_{2}^{i} & e_{3}^{i} \end{array}\right\}$, located at the center of mass of the *i*th rigid body with i∈N, and (ii) the inertial coordinate frame $E^{f}=\left\{\begin{array}{ccc} e_{1}^{f} & e_{2}^{f} & e_{3}^{f} \end{array}\right\}$, located at some point in the Earth’s surface, which for the sake of simplicity is assumed to be flat. This frame is typically chosen as the north-east-down (NED) frame with $e_{1}^{f}$ pointing to the north, $e_{2}^{f}$ pointing to the east and $e_{3}^{f}$ pointing to the center of Earth (see Figure 1). The rotation of the coordinates of a point from frame $E^{i}$ with respect to frame $E^{f}$ is represented by the attitude matrix $R\in SO\left(3\right)=\{R\in R^{3\times 3}:R^{T}R=I_{3},\det\left(R\right)=1\}$, where $I_{3}$ is the 3×3 identity matrix.

**Remark** **1.** *In this paper, $R_{i}^{f}$ is the matrix that rotates the coordinates of a point from frame $E^{i}$ to frame $E^{f}$.*

The cross product between two vectors $r,p\in {\mathbb{R}}^{3}$ is represented by a matrix multiplication $[r^{\times }]p=r\times p$, where $\left[r^{\times }\right]$ is the well known skew-symmetric matrix associated to vector r. The *n*-dimensional unit sphere embedded in ${\mathbb{R}}^{n+1}$ is denoted as ${𝕊}^{n}=\left\{x\in {\mathbb{R}}^{n+1}:x^{T}x=1\right\}$. Members of SO(3) are often parametrized in terms of a rotation $\beta^{i}\in \mathbb{R}$ about a fixed axis $e^{i}\in {𝕊}^{2}$ by the map $\mathbb{R}\times {𝕊}^{2}\to SO\left(3\right)$ defined as:

(2) $$R_{i}^{f}:=I_{3}+\sin\left(\beta^{i}\right)\left[{e^{i}}^{\times }\right]+\left(1-\cos\left(\beta^{i}\right)\right){\left[{e^{i}}^{\times }\right]}^{2}$$

The motion of the *i*th body-fixed reference frame $E^{i}$ relative to $E^{f}$ can be defined in terms of unit quaternion $q^{i}\in {𝕊}^{3}$, that is defined as:

(3) $$q_{i}:=\left(\begin{array}{c} \cos\frac{\beta^{i}}{2}\\ e^{i}\sin\frac{\beta^{i}}{2} \end{array}\right):=\left(\begin{array}{c} q_{i_{0}}\\ q_{i_{v}} \end{array}\right)\in {𝕊}^{3}$$

where $q_{i_{v}}={\left(\begin{array}{ccc} q_{i_{1}} & q_{i_{2}} & q_{i_{3}} \end{array}\right)}^{T}\in {\mathbb{R}}^{3}$ and $q_{i_{0}}\in \mathbb{R}$ are known as the vector and scalar parts of the quaternion respectively. Furthermore, $q_{i}$ represents an element of SO(3) through the map $R_{i}^{f}:{𝕊}^{3}\to SO\left(3\right)$ defined as:

(4) $$R_{i}^{f}\left(q_{i}\right):=I_{3}+2q_{i_{0}}\left[q_{i_{v}}^{\times }\right]+2{\left[q_{i_{v}}^{\times }\right]}^{2}$$

Note that $R_{i}^{f}\left(q_{i}\right)=R_{i}^{f}\left(-q_{i}\right)$ for each $q_{i}\in {𝕊}^{3}$, i.e., quaternions $q_{i}$ and $-q_{i}$ represent the same physical attitude. Let $\omega_{i}={\left(\begin{array}{ccc} \omega_{i_{1}} & \omega_{i_{2}} & \omega_{i_{3}} \end{array}\right)}^{T}\in {\mathbb{R}}^{3}$ be the angular velocity vector of the body coordinate frame $E^{i}$ relative to the inertial coordinate frame $E^{f}$ expressed in $E^{i}$. Then, the kinematics equation is given by:

(5) $${\dot{q}}_{i}=\frac{1}{2}\left(\begin{array}{c} -q_{i_{v}}^{T}\\ I_{3}q_{i_{0}}+\left[q_{i_{v}}^{\times }\right] \end{array}\right)\omega_{i}:=\frac{1}{2}\Xi \left(q_{i}\right)\omega_{i}$$

The attitude error is used to quantify the mismatch between two attitudes. If $q_{i}$ defines the current attitude quaternion and $q_{i}^{d}$ the desired quaternion, i.e., the desired orientation, then the quaternion that represents the attitude error between the current orientation and the desired one is given by:

(6) $${\tilde{q}}_{i}={\left(q_{i}^{d}\right)}^{-1}⊙q_{i}={\left(\begin{array}{cc} {\tilde{q}}_{i_{0}} & {\tilde{q}}_{i_{v}}^{T} \end{array}\right)}^{T}$$

where $q^{-1}$ is the complementary rotation of the quaternion q, which is given by $q^{-1}={\left(\begin{array}{cc} q_{0} & -q_{v}^{T} \end{array}\right)}^{T}$ and ⊙ denotes the quaternion multiplication [42]. When the current quaternion $q_{i}$ reaches the desired one $q_{i}^{d}$, the quaternion error becomes ${\tilde{q}}_{i}={\left(\begin{array}{cc} \pm 1 & 0^{T} \end{array}\right)}^{T}$. Remember that a quaternion has two equilibria (i.e., $q_{i}$ and $-q_{i}$) and this is considered in the stability analysis [43].

**Remark** **2.** *Euler angles can also be used for attitude representation, i.e., let $\phi_{i}$, $\theta_{i}$ and $\psi_{i}$ be the roll, pitch and yaw angles of the ith rigid body respectively. Typically, Euler angles will be used in the present paper to obtain a virtual control used like a bridge between the attitude control and the position control of the vehicles. Therefore, a rotation matrix is needed. The one through the map $R\left(\phi_{i},\theta_{i},\psi_{i}\right):{\mathbb{R}}^{3}\to SO\left(3\right)$ is defined by:*

(7) $$R_{f}^{i}\left(\Theta \right):=R\left(\phi_{i},\theta_{i},\psi_{i}\right)=\left(\begin{array}{ccc} C\psi_{i}\;C\theta_{i} & S\psi_{i}\;C\theta_{i} & -S\theta_{i}\\ C\psi_{i}\;S\theta_{i}\;S\phi_{i}-S\psi_{i}\;C\theta_{i} & S\phi_{i}\;S\theta_{i}\;S\psi_{i}+C\psi_{i}\;C\phi_{i} & C\theta_{i}\;S\phi_{i}\\ C\psi_{i}\;C\phi_{i}\;S\theta_{i}+S\psi_{i}\;S\phi_{i} & S\theta_{i}\;S\psi_{i}\;C\phi_{i}-C\psi_{i}\;S\phi_{i} & C\theta_{i}\;C\phi_{i} \end{array}\right)$$

*where C(·) and S(·) denote the sine and cosine functions for ease of reading. Note that the rotation matrix (7) describes the rotation from the inertial frame to the body-fixed one.*

#### 2.4.2. VTOL-UAVs Model

**Figure 2:**
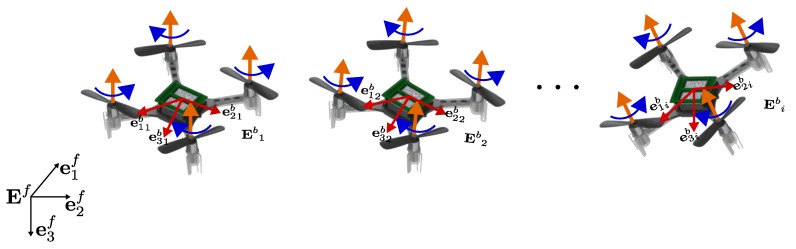
Group of *N* VTOL-UAVs.

Consider a group of *N* four-rotor helicopters, also called *quadcopter* hereafter (see Figure 2). According to the details mentioned before and to [44], the six degrees-of-freedom model (position and attitude) for each VTOL-UAV agent i∈N={1,…,N} can be separated into translational and rotational motions, i.e., $\Sigma_{T_{i}}$ and $\Sigma_{R_{i}}$ respectively, as follows:

(8) $$\begin{array}{ccc} \Sigma_{T_{i}} & : & \left\{\begin{array}{c} {\dot{p}}_{i}=v_{i}\\ {\dot{v}}_{i}=ge_{3}^{f}-\frac{1}{m_{i}}R_{i}^{f}e_{3}^{i}T_{i} \end{array}\right. \end{array}$$

(9) $$\begin{array}{ccc} \Sigma_{R_{i}} & : & \left\{\begin{array}{c} {\dot{q}}_{i}=\frac{1}{2}\Xi \left(q_{i}\right)\omega_{i}\\ J_{i}{\dot{\omega }}_{i}=-\left[\omega_{i}^{\times }\right]J_{i}\omega_{i}+\Gamma_{i} \end{array}\right. \end{array}$$

where $m_{i}$ is the mass of the *i*th quadcopter and $J_{i}$ its inertial matrix expressed in $E^{i}$. *g* is the mass acceleration and $e_{3}^{i}=e_{3}^{f}={\left(\begin{array}{ccc} 0 & 0 & 1 \end{array}\right)}^{T}$. $p_{i}\in {\mathbb{R}}^{3}$ denotes the position of the vehicle’s center of mass, which coincides with the origin of frame $E^{i}$ with respect to frame $E^{f}$. $v_{i}\in {\mathbb{R}}^{3}$ is its linear velocity in $E^{f}$, and $\omega_{i}\in {\mathbb{R}}^{3}$ the angular velocity vector of the body coordinate frame $E^{i}$ relative to the inertial coordinate frame $E^{f}$ expressed in $E^{i}$. The input $\Gamma_{i}\in {\mathbb{R}}^{3}$ represents the couples generated by the actuators, aerodynamic couples and external couples (environmental forces). It is typically assumed that these torques are only generated by actuators. The positive scalar $T_{i}$ denotes the *i*th vehicle’s total thrust applied to the airframe by the four rotors in the direction of $e_{3}^{i}$.

## 3. Attitude and Position (Inner) Control Loop

Previously, Section 2 described the agent model in the group of *N* controlled VTOL-UAVs. Now, the objective is to stabilize each agent independently, in a first inner control loop. Finally, a second outer control loop will be detailed in Section 4 to drive all the agents to a given consensus or formation.

### 3.1. Attitude Control

Note that (8) and (9) constitute a cascade system. For each VTOL-UAV *i*, with 1≤i≤N, $\Sigma_{T_{i}}$ in (8) represents the translational dynamics which depends on $\Sigma_{R_{i}}$, whereas $\Sigma_{R_{i}}$ in (9) represents the rotational dynamics which does not depend on $\Sigma_{T_{i}}$. Consequently, $\Gamma_{i}$ will be independently designed in a first step.

**Definition** **1.** *Given a positive constant M, a continuous and nondecreasing (saturation) function $\sigma_{M}:\mathbb{R}\to \mathbb{R}$ is defined by:*

(10) $$\sigma_{M}\left(s\right)=\left\{\begin{array}{cc} s & \mathrm{if}\;|s|<M\\ \mathrm{sign}(s)M & \mathrm{elsewhere} \end{array}\right.$$

**Theorem** **1.** *Consider the ith vehicle’s rotational dynamics described by (9) and the attitude error defined in (6), with the following bounded control inputs $\Gamma_{i_{l}}={\left(\begin{array}{ccc} \Gamma_{i_{l}} & \Gamma_{i_{2}} & \Gamma_{i_{3}} \end{array}\right)}^{T}$, such that*

(11) $$\Gamma_{i_{l}}=-\sigma_{M_{i}^{l}}\left(\frac{\kappa_{i}\omega_{i_{l}}}{\rho_{i_{l}}}+\kappa_{i}{\tilde{q}}_{i_{l}}\right)$$

*where $\sigma_{M_{i}^{l}}\left(\cdot \right)$ are saturation functions as defined in (10), with l∈{1,2,3}. $M_{i}^{l}$ represents the physical bound on the $l^{th}$ torque of the ith vehicle. $\kappa_{i}$ is a real parameter such that $0<\kappa_{i}\leq \min_{l}M_{i}^{l}/2$. $\rho_{i_{l}}$ are strictly positive real parameters. Then, inputs (11) asymptotically stabilize the VTOL-UAV quadcopters to the desired attitude $q_{i}^{d}$ (i.e., ${\tilde{q}}_{i_{0}}=1$, ${\tilde{q}}_{i_{v}}=0$, $\omega_{i}=0$) with a domain of attraction for the attitude error and angular velocity equal to ${𝕊}^{3}\times {\mathbb{R}}^{3}\setminus {\left(\begin{array}{ccc} -1 & 0^{T} & 0^{T} \end{array}\right)}^{T}$.*

**Proof.**  The proof follows the one presented in [44]. □

### 3.2. Position Control

Once the rotational dynamics $\Sigma_{R_{i}}$ is stabilized in the cascade system (8) and (9), the control for the translational dynamics $\Sigma_{T_{i}}$ can be handled. The objective is to design a control strategy which stabilizes a VTOL-UAV to a certain position in the space. For that, consider (8) where the matrix rotation $R_{i}^{f}$ is parameterized using the Euler angles as in (7). Note that $R_{i}^{f}={\left(R_{f}^{i}\right)}^{T}$. Assume that the yaw dynamics of the *i*th VTOL-UAV can be stabilized by using the control law (11), that yields $\psi_{i}\to 0$. Then, when the yaw vanishes, the relation (8) becomes:

(12) $$\begin{array}{ccc} \Sigma_{1_{i}} & : & \left\{\begin{array}{c} {\dot{p}}_{i_{1}}=v_{i_{1}}\\ {\dot{v}}_{i_{1}}=-\frac{T_{i}}{m_{i}}\cos\phi_{i}\sin\theta_{i} \end{array}\right. \end{array}$$

(13) $$\begin{array}{ccc} \Sigma_{2_{i}} & : & \left\{\begin{array}{c} {\dot{p}}_{i_{2}}=v_{i_{2}}\\ {\dot{v}}_{i_{2}}=\frac{T_{i}}{m_{i}}\sin\phi_{i} \end{array}\right. \end{array}$$

(14) $$\begin{array}{ccc} \Sigma_{3_{i}} & : & \left\{\begin{array}{c} {\dot{p}}_{i_{3}}=v_{i_{3}}\\ {\dot{v}}_{i_{3}}=-\frac{T_{i}}{m_{i}}\cos\phi_{i}\cos\theta_{i}+g \end{array}\right. \end{array}$$

As for the yaw angle, we have to choose an appropriate target attitude, such that thanks to control law (11), the systems (12)–(14) will be able to be transformed into three double integrator subsystems [45,46]. Indeed, consider the angle references defined by:

(15) $$\begin{array}{cc} \; & \theta_{d_{i}}:=\arctan\left(\frac{r_{i_{1}}}{r_{i_{3}}-g}\right),\\ \; & \phi_{d_{i}}:=\arctan\left(\frac{r_{i_{2}}}{\sqrt{r_{i_{1}}^{2}+{\left(r_{i_{3}}-g\right)}^{2}}}\right) \end{array}$$

$r_{i_{1}},r_{i_{2}},r_{i_{3}}$ will be suitably designed to achieve collaborative control in the next section. Consider also the positive thrust as:

(16) $$T_{i}=m_{i}\sqrt{r_{i_{1}}^{2}+r_{i_{2}}^{2}+{\left(r_{i_{3}}-g\right)}^{2}}$$

Then, after a sufficiently long time, $\theta_{i}=\theta_{d_{i}}$ and $\phi_{i}=\phi_{d_{i}}$. Note that $\theta_{i}$ and $\phi_{i}$ represent the (unique) angle such that:

(17) $$\begin{array}{ccc} \sin\phi_{i} & = & \frac{r_{i_{2}}}{\sqrt{r_{i_{1}}^{2}+r_{i_{2}}^{2}+{\left(r_{i_{3}}-g\right)}^{2}}} \end{array}$$

(18) $$\begin{array}{ccc} \cos\phi_{i} & = & \frac{\sqrt{r_{i_{1}}^{2}+{\left(r_{i_{3}}-g\right)}^{2}}}{\sqrt{r_{i_{1}}^{2}+r_{i_{2}}^{2}+{\left(r_{i_{3}}-g\right)}^{2}}} \end{array}$$

(19) $$\begin{array}{ccc} \sin\theta_{i} & = & \frac{-r_{i_{1}}}{\sqrt{r_{i_{1}}^{2}+{\left(r_{i_{3}}-g\right)}^{2}}} \end{array}$$

(20) $$\begin{array}{ccc} \cos\theta_{i} & = & \frac{-(r_{i_{1}}-g)}{\sqrt{r_{i_{1}}^{2}+{\left(r_{i_{3}}-g\right)}^{2}}} \end{array}$$

Substituting (16) and (20) in (12) and (14) gives three independent linear double integrator subsystems:

(21) $$\begin{array}{ccc} \Sigma_{1_{i}}^{T} & : & \left\{\begin{array}{c} {\dot{p}}_{i_{1}}=v_{i_{1}}\\ {\dot{v}}_{i_{1}}=r_{i_{1}} \end{array}\right. \end{array}$$

(22) $$\begin{array}{ccc} \Sigma_{2_{i}}^{T} & : & \left\{\begin{array}{c} {\dot{p}}_{i_{2}}=v_{i_{2}}\\ {\dot{v}}_{i_{2}}=r_{i_{2}} \end{array}\right. \end{array}$$

(23) $$\begin{array}{ccc} \Sigma_{3_{i}}^{T} & : & \left\{\begin{array}{c} {\dot{p}}_{i_{3}}=v_{i_{3}}\\ {\dot{v}}_{i_{3}}=r_{i_{3}} \end{array}\right. \end{array}$$

**Remark** **3.** *The design of the virtual control inputs $r_{i_{1}}$, $r_{i_{2}}$ and $r_{i_{3}}$ is considered in a framework of cooperative control in the next section.*

## 4. Distributive Event-Triggered Protocol for Consensus and Formation

Once the attitude control problem is solved for one quadcopter, the objective is to exploit the underactuated nature of VTOL-UAVs to design a control law which stabilizes the position of multiple VTOL-UAVs (as represented in Figure 2) to a specific point in space (consensus problem) or a particular shape (formation problem).

### 4.1. Leader-Following Consensus Control

Here, the design of a control strategy for *N* quadcopters, described by (8) and (9), is addressed. Also, consider that control signals $\Gamma_{i}$ in (11) as well as $T_{i}$ in (16) and $\theta_{d_{i}}$, $\phi_{d_{i}}$ in (15) are applied in an inner control loop in each vehicle i∈V. Therefore, each VTOL-UAV *i* can be modeled as in (21)–(23), whose virtual control inputs $r_{i_{1}}$, $r_{i_{2}}$ and $r_{i_{3}}$ will be synthesized for leader-following consensus control purposes.

Let us establish the following state variables: $\xi_{i_{1}}=p_{i_{1}}$, $\xi_{i_{2}}=v_{i_{1}}$, $\xi_{i_{3}}=p_{i_{2}}$, $\xi_{i_{4}}=v_{i_{2}}$, $\xi_{i_{5}}=p_{i_{3}}$, $\xi_{i_{6}}=v_{i_{3}}$. Then, system (12)–(14) can be rewritten as:

(24) $$\dot{\xi_{i}}=\bar{A}\xi_{i}+\bar{B}{\bar{u}}_{i}$$

where $\xi_{i}={\left(\begin{array}{cccccc} \xi_{i_{1}} & \xi_{i_{2}} & \xi_{i_{3}} & \xi_{i_{4}} & \xi_{i_{5}} & \xi_{i_{6}} \end{array}\right)}^{T}$ is the state vector for the *i*th VTOL-UAV, ${\bar{u}}_{i}={\left(\begin{array}{ccc} r_{i_{1}} & r_{i_{2}} & r_{i_{3}} \end{array}\right)}^{T}$ is its control input, $\bar{A}\in {\mathbb{R}}^{6\times 6}$ and $\bar{B}\in {\mathbb{R}}^{6\times 3}$. Note that the pair $\left(\bar{A},\bar{B}\right)$ is stabilizable. Furthermore, let us define the virtual leader’s dynamics, which is labeled 0 as follows:

(25) $$\dot{\xi_{0}}={\bar{A}}_{0}\xi_{0}$$

with $\xi_{0}\in {\mathbb{R}}^{6}$ and ${\bar{A}}_{0}\in {\mathbb{R}}^{6\times 6}$. Actually, the virtual leader VTOL-UAV (25) acts like an exosystem which provides the requested target reference or trajectory. Then, a first objective is to design local controllers ${\bar{u}}_{i}$ for all follower nodes (24). A second objective is to propose a triggering rule to determine, using only local information, the instant when the *i*th quadcopter (or agent) has to communicate a new state value to its neighbors.

**Definition** **2.** *Consider the system (24) and (25). It is said that the consensus is practically achieved, using the event-triggered protocol* ${\bar{u}}_{i}$ *for each vehicle* i∈V *, if the closed-loop system satisfies:*

(26) $$\lim_{t\to \infty }∥\xi_{i}\left(t\right)-\xi_{0}\left(t\right)∥=\Delta \;i=1,\ldots ,N$$

*for any initial condition* $\xi_{0}\left(0\right)$ *and* $\xi_{i}\left(0\right)$ *, where* $\Delta \in {\mathbb{R}}_{+}$ *.*

Then, the main proposal follows:

**Theorem** **2.** *Let (24) and (25) be the multi-vehicle system which is subject to the following control strategy:*

(27) $${\bar{u}}_{i}=K\left[\sum_{j\in N_{i}}\left(m_{j}-m_{i}\right)+g_{i}\left(\xi_{0}-m_{i}\right)\right]$$

*with*$K=\rho {\bar{B}}^{T}P$*and*$g_{i}$*the pinning gains (*$g_{i}=1$*if the node i observes the leader and*$g_{i}=0$*otherwise), where P is a positive definite matrix solution to the Riccati equation:*

$${\bar{A}}^{T}P+P\bar{A}-2\rho P\bar{B}{\bar{B}}^{T}P=-Q$$

*Q is also positive definite and*ρ>0*. Suppose the event function is given by:*

(28) $$\epsilon_{i}\left(\xi_{i},m_{i}\right)={\tilde{e}}_{i_{1}}\wedge {\tilde{e}}_{i_{2}}\wedge {\tilde{e}}_{i_{3}}\wedge {\tilde{e}}_{i_{4}}\wedge {\tilde{e}}_{i_{5}}\wedge {\tilde{e}}_{i_{6}}$$

*where the symbol ∧ denotes the "or" logical connective. Moreover:*

(29) $${\tilde{e}}_{i_{s}}=\left\{\begin{array}{cc} 1 & \mathrm{if}\;\left|m_{i_{s}}-\xi_{i_{s}}\right|-\delta \geq 0\\ 0 & \mathrm{elsewhere} \end{array}\right.$$

*where*$\delta \in {\mathbb{R}}_{+}$*and*s={1,2,3,4,5,6}*for each state in (24) and (25). Then, from any initial condition, all vehicles follow the virtual leader and converge to a neighborhood given by:*

(30) $$\lim_{t\to \infty }∥\tilde{\xi }\left(t\right)∥=\frac{\sqrt{6N}\delta ∥\tilde{B}∥\bar{\alpha }}{\lambda_{1}^{\tilde{A}}}=\Delta$$

*where*$\tilde{\xi }={\left(\begin{array}{cccc} {\tilde{\xi }}_{1} & {\tilde{\xi }}_{2} & \ldots & {\tilde{\xi }}_{N} \end{array}\right)}^{T}$*and*${\tilde{\xi }}_{i}=\xi_{i}-\xi_{0}$*,*i∈V*. Furthermore,*$\lambda_{1}^{\tilde{A}}=\lambda_{\min}\left(\tilde{A}\right)$*being*$\tilde{A}=I_{N}⊗\bar{A}-\tilde{B}$, $\bar{\alpha }=∥D∥∥D^{T}∥$*, with D a matrix used to diagonalize matrix*$\tilde{A}$. $\tilde{B}=-H⊗\bar{B}K$*, with*H=L+G*as introduced in Lemma 1.*

**Remark** **4.** *Note that* ${\bar{u}}_{i}$ *is a function of* $m_{i}$ *and* $m_{j}$ *, with* $j\in N_{i}$ *(node i’s neighbor set). As a remind, let* $m_{i}$ *be the latest broadcast state of vehicle i, i.e.,* $m_{i}\left(t\right)=\xi_{i}\left(t_{k}^{i}\right)$ *for* $t\in [t_{k}^{i},t_{k+1}^{i}[$ *, where* $t_{0}^{i},t_{1}^{i},\ldots$ *is the sequence of event times of agent i. Consequently,* $m_{j}$ *is the latest broadcast state of its neighbor.*

**Proof.**  Let ${\bar{e}}_{i}=m_{i}-\xi_{i}$ and ${\tilde{\xi }}_{i}=\xi_{i}-\xi_{0}$ be some error variables. Then, the control strategy (27) is rewritten as follows:

$${\bar{u}}_{i}=K\left[\sum_{j\in N_{i}}\left({\tilde{\xi }}_{j}-{\tilde{\xi }}_{i}\right)+g_{i}{\tilde{\xi }}_{i}+\sum_{j\in N_{i}}\left({\bar{e}}_{j}-{\bar{e}}_{i}\right)-g_{i}{\bar{e}}_{i}\right]$$

Substituting in (24), the closed-loop system becomes:

$$\begin{array}{ccc} {\dot{\tilde{\xi }}}_{i} & = & \bar{A}{\tilde{\xi }}_{i}+\bar{B}\left[K\sum_{j\in N_{i}}\left({\tilde{\xi }}_{j}-{\tilde{\xi }}_{i}\right)+Kg_{i}{\tilde{\xi }}_{i}+K\sum_{j\in N_{i}}\left({\bar{e}}_{j}-{\bar{e}}_{i}\right)-Kg_{i}{\bar{e}}_{i}\right]\\ & = & \bar{A}{\tilde{\xi }}_{i}+\bar{B}K\sum_{j\in N_{i}}\left({\tilde{\xi }}_{j}-{\tilde{\xi }}_{i}\right)+\bar{B}Kg_{i}{\tilde{\xi }}_{i}+\bar{B}K\sum_{j\in N_{i}}\left({\bar{e}}_{j}-{\bar{e}}_{i}\right)-\bar{B}Kg_{i}{\bar{e}}_{i} \end{array}$$

Defining $\tilde{\xi }={\left(\begin{array}{cccc} {\tilde{\xi }}_{1} & {\tilde{\xi }}_{2} & \ldots & {\tilde{\xi }}_{N} \end{array}\right)}^{T}$, $G=diag(g_{1},g_{2},\ldots ,g_{N})$, $\bar{e}={\left(\begin{array}{cccc} {\bar{e}}_{1} & {\bar{e}}_{2} & \ldots & {\bar{e}}_{N} \end{array}\right)}^{T}$ and by employing the Laplaciane L of the graph G, one has

(31) $$\dot{\tilde{\xi }}=\underset{\tilde{A}}{\underset{︸}{\left[\left(I_{N}⊗\bar{A}\right)-H⊗\bar{B}K\right]}}\tilde{\xi }+\underset{\tilde{B}}{\underset{︸}{\left[-H⊗\bar{B}K\right]}}\bar{e}$$

with H=L+G. Note that Lemma 1 establishes the properties of H.

Now, let’s first assume that $\bar{e}=0$, and one will show that the system $\dot{\tilde{\xi }}=\tilde{A}\tilde{\xi }$ is asymptotically stable. Let’s consider the following candidate Lyapunov function:

$$V\left(\tilde{\xi }\right)={\tilde{\xi }}^{T}\left(I_{N}⊗P\right)\tilde{\xi }$$

the derivative along the trajectories of (31) is:

$$\begin{array}{ccc} \dot{V}\left(\tilde{\xi }\right) & = & {\tilde{\xi }}^{T}\left[\left(I_{N}⊗{\bar{A}}^{T}\right)-\left(H⊗K^{T}{\bar{B}}^{T}\right)\left(I_{N}⊗P\right)\right]\tilde{\xi }+{\tilde{\xi }}^{T}\left[\left(I_{N}⊗P\right)\left(I_{N}⊗\bar{A}-H⊗\bar{B}K\right)\right]\tilde{\xi }\\ & = & {\tilde{\xi }}^{T}\left[I_{N}⊗\left(P\bar{A}+{\bar{A}}^{T}P\right)-H⊗\left(2P\bar{B}{\bar{B}}^{T}P\right)\right]\tilde{\xi } \end{array}$$

Because H is symmetric, one can find a matrix $T\in {\mathbb{R}}^{N\times N}$ such that $THT^{T}=\Lambda :=diag\left(\lambda_{1},\ldots ,\lambda_{N}\right)$ where $\lambda_{1},\ldots ,\lambda_{N}$ denote the eigenvalues of H which, according to Lemma 1, are positive.

Using the following linear transformation $\tilde{\tilde{\xi }}=\left(T⊗I_{N}\right)\tilde{\xi }$ one has:

$$\begin{array}{ccc} \dot{V} & = & {\tilde{\tilde{\xi }}}^{T}\left[\left(I_{N}⊗\left(P\bar{A}+{\bar{A}}^{T}P\right)\right)-\Lambda ⊗\left(2P\bar{B}{\bar{B}}^{T}P\right)\right]\tilde{\tilde{\xi }}\\ & \leq & \sum_{i=1}^{N}{\tilde{\tilde{\xi }}}_{i}^{T}\left[P\bar{A}+{\bar{A}}^{T}P-\lambda_{i}\left(2P\bar{B}{\bar{B}}^{T}P\right)\right]{\tilde{\tilde{\xi }}}_{i}\\ & \leq & -\sum_{i=1}^{N}{\tilde{\tilde{\xi }}}_{i}^{T}Q{\tilde{\tilde{\xi }}}_{i}\\ & \leq & -\sum_{i=1}^{N}{\tilde{\xi }}_{i}^{T}\underset{\bar{Q}}{\underset{︸}{\left(I_{N}⊗T^{T}\right)Q\left(T⊗I_{N}\right)}}{\tilde{\xi }}_{i}<0\;\forall \;{\tilde{\xi }}_{i}\neq 0 \end{array}$$

Therefore $\tilde{A}$ is Hurwitz and the error between followers and leader converges to zero, that is ${\tilde{\xi }}_{i}\to 0$ when $\bar{e}=0$ and t→∞.

Now, consider the case $\bar{e}\neq 0$ and assume that $\lambda_{1}^{\tilde{A}}=\lambda_{min}\left(\tilde{A}\right)$. Then the solution of (31), which can be written as follows:

(32) $$\dot{\tilde{\xi }}=\tilde{A}\tilde{\xi }+\tilde{B}\bar{e}$$

is given by:

$$\begin{array}{ccc} \tilde{\xi }\left(t\right) & = & e^{\tilde{A}t}\tilde{\xi }\left(0\right)+\int_{0}^{t}e^{\tilde{A}\left(t-\tau \right)}\tilde{B}\bar{e}\left(\tau \right)d\tau \\ ∥\tilde{\xi }\left(t\right)∥ & \leq & ∥e^{\tilde{A}t}\tilde{\xi }\left(0\right)∥+\int_{0}^{t}∥e^{\tilde{A}\left(t-\tau \right)}\tilde{B}\bar{e}\left(\tau \right)d\tau ∥ \end{array}$$

Moreover, let $D\tilde{A}D^{T}=\Phi =diag\left(\lambda_{1}^{\tilde{A}},\ldots ,\lambda_{N}^{\tilde{A}}\right)$ be a diagonal matrix, this results in:

$$\begin{array}{ccc} ∥\tilde{\xi }\left(t\right)∥ & \leq & \bar{\alpha }e^{-\lambda_{1}^{\tilde{A}}t}∥\tilde{\xi }\left(0\right)∥+\bar{\alpha }\int_{0}^{t}e^{-\lambda_{1}^{\tilde{A}}\left(t-\tau \right)}∥\tilde{B}\bar{e}\left(\tau \right)∥d\tau \end{array}$$

with

$$\bar{\alpha }=∥D∥∥D^{T}∥$$

As $∥\tilde{B}\bar{e}∥\leq ∥\tilde{B}∥∥\bar{e}∥$, and because of the event condition, one obtains:

$$∥\bar{e}∥\leq \sqrt{6\delta^{2}+6\delta^{2}+\ldots +6\delta^{2}}=\delta \sqrt{6N}$$

Consequently, the error (between followers and leader) is bounded as:

$$\begin{array}{ccc} ∥\tilde{\xi }\left(t\right)∥ & \leq & \bar{\alpha }e^{-\lambda_{1}^{\tilde{A}}t}∥\tilde{\xi }\left(0\right)∥+\bar{\alpha }\int_{0}^{t}e^{-\lambda_{1}^{\tilde{A}}\left(t-\tau \right)}2\delta \sqrt{N}∥\tilde{B}∥d\tau \\ & \leq & \bar{\alpha }e^{-\lambda_{1}^{\tilde{A}}t}∥\tilde{\xi }\left(0\right)∥+2\bar{\alpha }e^{-\lambda_{1}^{\tilde{A}}t}∥\tilde{B}∥\delta \sqrt{N}\int_{0}^{t}e^{\lambda_{1}^{\tilde{A}}\tau }d\tau \\ & \leq & \bar{\alpha }e^{-\lambda_{1}^{\tilde{A}}t}∥\tilde{\xi }\left(0\right)∥+2\bar{\alpha }e^{-\lambda_{1}^{\tilde{A}}t}∥\tilde{B}∥\delta \sqrt{N}\left(\frac{e^{\lambda_{1}^{\tilde{A}}t}}{\lambda_{1}^{\tilde{A}}}-\frac{1}{\lambda_{1}^{\tilde{A}}}\right) \end{array}$$

Then

(33) $$\begin{array}{ccc} ∥\tilde{\xi }\left(t\right)∥ & \leq & \frac{\bar{\alpha }\delta \sqrt{6N}∥\tilde{B}∥}{\lambda_{1}^{\tilde{A}}}=\Delta \end{array}$$

Then, the proposed event-triggered distributive control strategy allows achieving practically a leader-following consensus, i.e., the error between the followers and the leader converges to a ball centered at the origin with radius Δ. □

### 4.2. Exclusion of Zeno Behavior

In order to exclude Zeno behavior, we show that the inter-event time is lower bounded by a positive constant. For this, let us analyze the behavior of ${\bar{e}}_{i}=m_{i}-\xi_{i}$ just after an event has taken place. The system’s global vectors can be defined as $\bar{e}=m-\xi \in {\mathbb{R}}^{6N}$, $\tilde{\xi }=\xi -{\bar{\xi }}_{0}\in {\mathbb{R}}^{6N}$, where ${\bar{\xi }}_{0}={\left(\xi_{0}^{T},\xi_{0}^{T},\cdots \xi_{0}^{T}\right)}^{T}\in {\mathbb{R}}^{N6}$. Then, $\bar{e}=m-\left(\tilde{\xi }+{\bar{\xi }}_{0}\right)$, hence:

(34) $$\dot{\bar{e}}\left(t\right)=\dot{m}-\dot{\tilde{\xi }}\left(t\right)-{\dot{\bar{\xi }}}_{0}\left(t\right)$$

From (25) and (32), one obtains:

(35) $$\begin{array}{ccc} \dot{\bar{e}}\left(t\right) & = & -\left(\tilde{A}\tilde{\xi }+\tilde{B}\bar{e}\right)-\left(I_{N}⊗{\bar{A}}_{0}\right){\bar{\xi }}_{0} \end{array}$$

(36) $$\begin{array}{ccc} & = & -\tilde{A}\left(m-\bar{e}-{\bar{\xi }}_{0}\right)-\tilde{B}\bar{e}-\left(I_{N}⊗{\bar{A}}_{0}\right){\bar{\xi }}_{0} \end{array}$$

(37) $$\begin{array}{ccc} & = & \left(\tilde{A}-\tilde{B}\right)\bar{e}+\tilde{A}\left({\bar{\xi }}_{0}-m\right)-\left(I_{N}⊗{\bar{A}}_{0}\right){\bar{\xi }}_{0} \end{array}$$

Let ${\bar{\gamma }}_{1},{\bar{\gamma }}_{2},{\bar{\gamma }}_{3}\in {\mathbb{R}}_{+}$ be the upper bounds of $\tilde{A}-\tilde{B}$, $\tilde{A}\left({\bar{\xi }}_{0}-m\right)$, and $\left(I_{N}⊗{\bar{A}}_{0}\right){\bar{\xi }}_{0}$, respectively. Then, it follows that

(38) $$∥\dot{\bar{e}}\left(t\right)∥\leq {\bar{\gamma }}_{1}∥\bar{e}\left(t\right)∥+{\bar{\gamma }}_{2}+{\bar{\gamma }}_{3}$$

Similar reasoning can be used to split the global vector $\dot{\bar{e}}$ in each ${\bar{e}}_{i}$, as follows:

(39) $$∥{\dot{\bar{e}}}_{i}\left(t\right)∥\leq \gamma_{1}∥{\bar{e}}_{i}\left(t\right)∥+\gamma_{2}+\gamma_{3}$$

with $\gamma_{1},\gamma_{2},\gamma_{3}\in {\mathbb{R}}_{+}$. Note that (39) is a first order differential equation with initial condition $∥{\bar{e}}_{i}\left(t_{k}^{i}\right)∥$. Note that just after an event is triggered for the agent *i*, ${\bar{e}}_{i}\left(t_{k}^{i}\right)=0$. Define $r=t-t_{k}^{i}$, then $r\in [0,t_{k+1}^{i}-t_{k}^{i}[$, and the solution of (39) with $\gamma_{T}=\gamma_{1}+\gamma_{2}$ is:

(40) $$∥{\bar{e}}_{i}\left(t\right)∥=∥{\bar{e}}_{i}\left(r+t_{k}^{i}\right)∥=\int_{0}^{r}e^{\gamma_{2}\left(r-\tau \right)}\gamma_{T}d\tau =\frac{\gamma_{T}}{\gamma_{2}}\left(e^{\gamma_{2}\left(t-t_{k}^{i}\right)}-1\right)$$

According to (28) and (29) the next event will be triggered as soon as $\left|m_{i_{s}}-\xi_{i_{s}}\right|\geq \delta$, i.e., when $∥{\bar{e}}_{i}\left(t\right)∥\geq \delta$, it follows that:

(41) $$\frac{\gamma_{T}}{\gamma_{2}}\left(e^{\gamma_{2}\left(t-t_{k}^{i}\right)}-1\right)\geq \delta$$

consequently, a lower bound on the inter-event times is given by:

(42) $$\left(t-t_{k}^{i}\right)\geq \frac{1}{\gamma_{2}}\ln\left(\frac{\gamma_{2}}{\gamma_{T}}\delta +1\right)$$

then Zeno phenomena like accumulation points are avoided since it is possible to ensure that there is a minimal sampling time between two consecutive events for all agents *i*.

### 4.3. Formation Control

In the case of formation control, one aims to obtain geometrical patterns to be accomplished by the group of vehicles. The control strategy (27) is naturally extended to leader-following formation. The objective is that followers move forward a virtual leader’s target reference while preserving the desired form.

Define Υ, as a collection of relative desired inter-agent distances, that is:

(43) $$\Upsilon =\left\{\varrho_{ij}\in \mathbb{R}|\varrho_{ij}>0,i,j=1,\ldots ,N,i\neq 0\right\}$$

with $\varrho_{ij}=\varrho_{ji}$. Let the formation be defined by a specification given through an associated target location set *F* given by

(44) $$F=\left\{\zeta_{1},\zeta_{2},\ldots ,\zeta_{N}\right\},\;\zeta_{i}\in {\mathbb{R}}^{6},\;i=1,\ldots ,N$$

where

(45) $$∥\zeta_{i}-\zeta_{j}∥=\varrho_{ij}$$

**Remark** **5.** *The follower agents must perform a target reference with final zero velocity (comparable to the regulation case), then, second, fourth, and sixth components of vectors $\zeta_{i}$ and $\zeta_{j}$ have to be zero,*

Remember that $\xi_{i}\in {\mathbb{R}}^{6}$ denotes the agent’s state. The objective by the formation protocol is that for some $\bar{\tau }\in {\mathbb{R}}^{6},\xi_{i}=\zeta_{i}+\bar{\tau }$, for all i=1,2,…,n. Again, since the agents must perform a target reference with final zero velocity the second, fourth, and sixth components of vectors $\bar{\tau }$ have to be zero.

In this way, the consensus protocol is extended to the formation one, if the desired shape is described through an associated target location set *F*. In this case, the control strategy (27) becomes:

(46) $${\bar{u}}_{i}=K\left[\sum_{j\in N_{i}}\left(m_{j}-m_{i}\right)-\left(\zeta_{j}-\zeta_{i}\right)+g_{i}\left(\xi_{0}-m_{i}\right)\right]$$

with such a protocol one guarantees that:

1. $\lim_{t\to \infty }∥\xi_{i}\left(t\right)-\xi_{0}\left(t\right)∥=\Delta$ with i=1,…,N
2. $\lim_{t\to \infty }∥\xi_{i}\left(t\right)-\xi_{j}\left(t\right)∥=\varrho_{ij}$. Note that i,j are such that (i,j)∈E and (j,i)∈E for the unidirected graph G={V,E}.

## 5. Numerical and Experimental Tests

**Figure 3:**
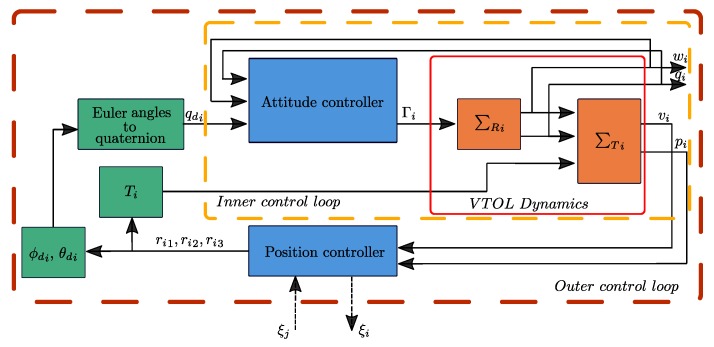
Event-triggered control strategy.

In the present section, the aim is to validate the proposed control strategies via simulations and real-time physical implementation. Considering as VTOL-UAVs a set of nano-quadcopters, which are modeled as in (8) and (9). Figure 3 describes the control strategy operating in the *i*th vehicle. The results are separated below in numerical simulations and experimental tests.

### 5.1. Simulation Tests

**Figure 4:**
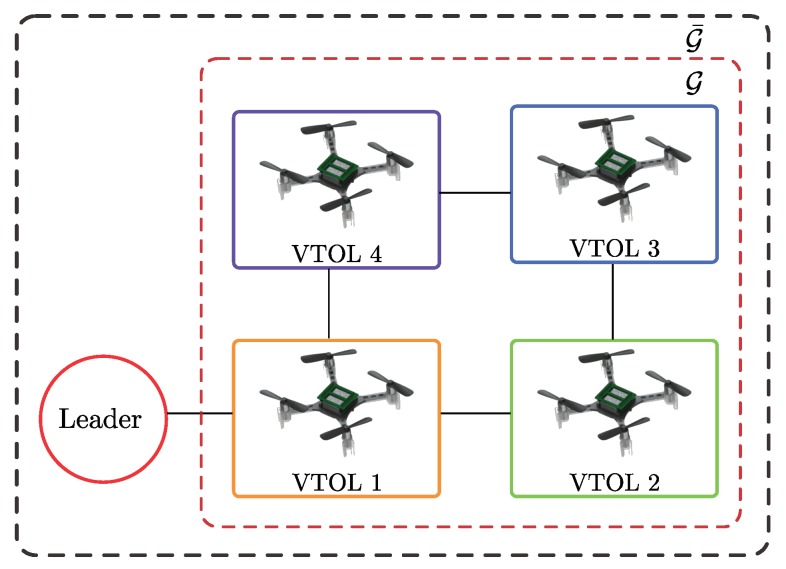
Graph for simulation results.

The control strategy has been validated via a collection of numerical simulations. Four agents are considered, where each agent of the collaborative system is represented by a VTOL-UAV. The closed-loop system, i.e.: the mathematical model of the VTOL-UAV and the event-triggered cooperative control strategy are implemented in the *Matlab/Simulink* environment. The communication topology between agents is considered as the undirected graph G which is shown in Figure 4, where VTOL 1 is the only agent receiving information from the virtual leader. The simulations aim to confirm that the control strategy drives each of the agents to the solicited consensus and formation. The desired formation refers to given positions that each of the agents has to attain and maintain. Two scenarios are presented:

1. The first scenario shows the consensus of four agents pursuing reference positions provided by the leader;
2. A second one shows the evolution of the collaborative system for consensus and formation control. Moreover, the robustness to an external perturbation on one of the agents is illustrated.

#### 5.1.1. Scenario One

**Figure 5:**
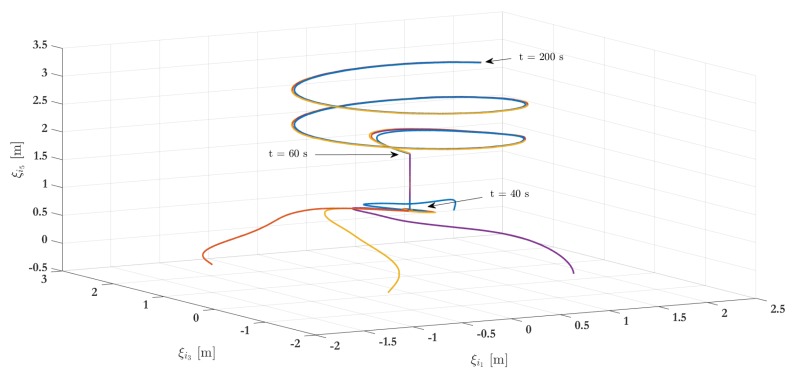
Simulation results—stage one: 3D representation.

**Figure 6:**
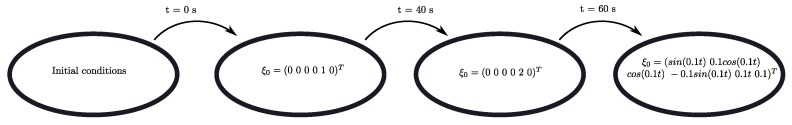
Simulation results—stage one: simulation flowchart.

The numerical simulation results of the first scenario addresses three arrangements integrated by four agents for 200 s. Figure 5 shows the position of all agents in a 3D representation, where the vehicles trajectories are highlighted in the plot (in dot line) and the time when all agents achieve the different stages is also depicted. The initial attitude and position for each vehicle are depicted in Table 1, with zero angular and linear velocities. In this simulation, the UAVs start from initial condition, and the leader-following consensus protocol (27) is executed being $\xi_{0}={\left(0\;0\;0\;0\;1\;0\right)}^{T}$ the virtual leader position, i.e., the reference point for the followers. Once the consensus has been obtained at the fixed reference point $\xi_{0}$ (which is achieved at t = 40 s), the virtual leader switches to be $\xi_{0}={\left(0\;0\;0\;0\;2\;0\right)}^{T}$ and the consensus is achieved at t=60 s. Then, $\xi_{0}$ is parameterized in time as $\xi_{0}={\left(\sin\left(0.1t\right)\;0.1\cos\left(0.1t\right)\;\cos\left(0.1t\right)\;-0.1\sin\left(0.1t\right)\;0.01t\;0.1\right)}^{T}$ for t>60 s. Meanwhile, the consensus protocol (27) continues to be executed. Figure 6 shows the three main phases during the simulation.

**Figure 7:**
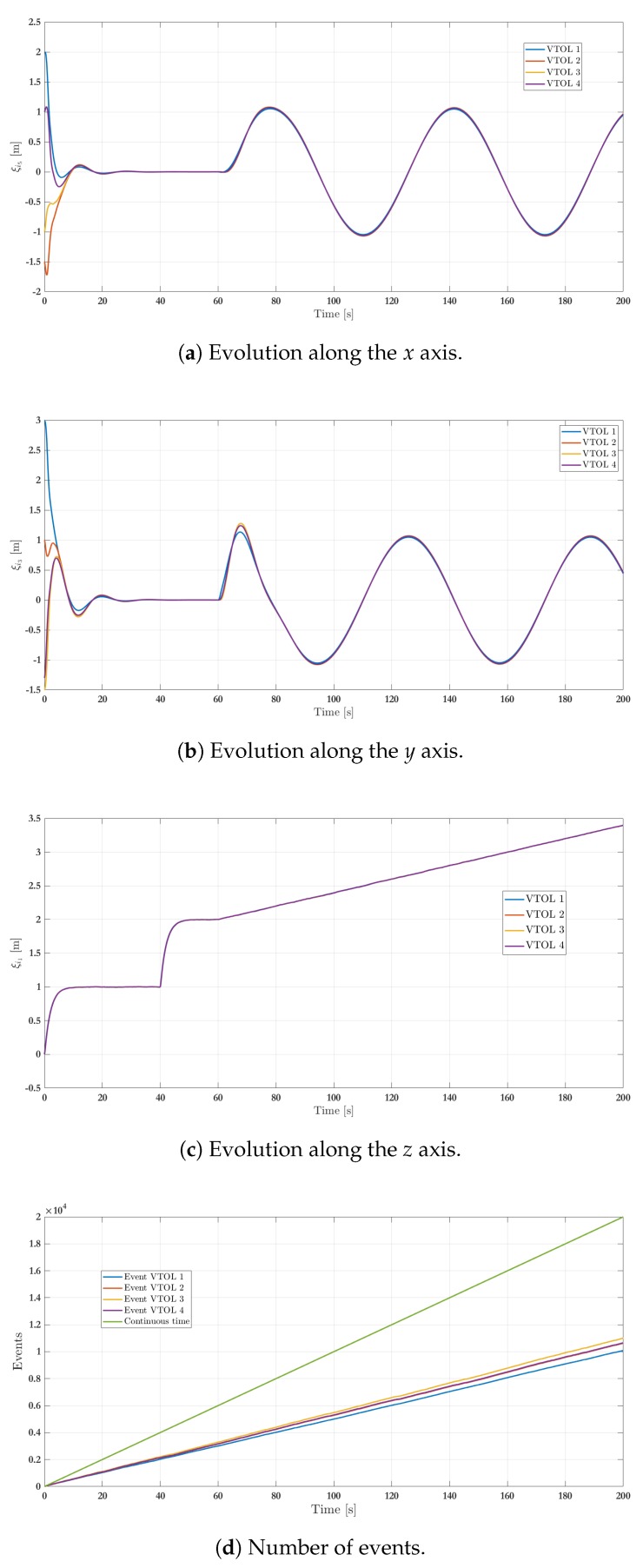
Simulation results—stage one: VTOL-UAV positions.

Figure 7a–c show the position of agents, represented by $\left(\xi_{i_{1}},\xi_{i_{3}},\xi_{i_{5}}\right)$ respectively, through time. Remark that every quadcopter starts from its initial condition and reaches the expected consensus. The numerical results consider a transmission sampling rate among agents of 0.01 s. When an event occurs for a given agent, its state value is transmitted to the closest neighbors (determined by the graph in Figure 4). Figure 7d shows the amount of events generated for the control strategy during the simulation. Typically, the slope of events increases a lot during transients (when an agent has to reach a new arrangement) while it only increases slightly during steady state intervals. The plot also shows a comparison with respect to the continuous-time transmission scheme, where events periodically occur. Whereas the behavior seems similar at the beginning, because the system is moving from an initial state to a desired formation, there is a clear reduction on the transmission rate of events after about 10 s, i.e., when steady-state behavior is reached. Indeed, considering a 0.01 s transmission rate and a 200 s simulation time, then the total amount of transmitted events between neighbors is 20,000 for each agent in the periodic case. With the proposed event-triggered control strategy, the amount of transmissions is reduced to 10,080 for VTOL 1, 10,630 for VTOL 2, 11,010 for VTOL 3 and 10,660 for VTOL 4. This represents a clear benefit to reduce the usage of communication bandwidth in the system and, therefore, in resource consumption, about 50.4%, 53.15%, 55.05%, and 53.3%, respectively. A video of this scenario can be viewed in the following link: Video of Scenario 1 (https://www.dropbox.com/s/844r26x4y2gikgd/Consenso_MDPI_2019.mp4?dl=0).

#### 5.1.2. Scenario Two

**Figure 8:**
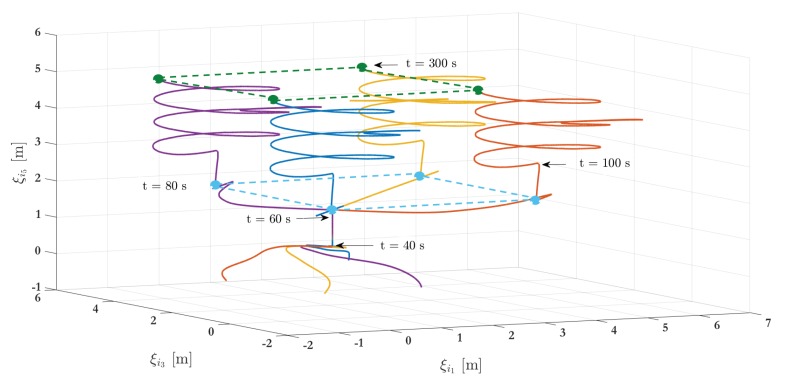
Simulation results—stage two: 3D representation.

**Figure 9:**
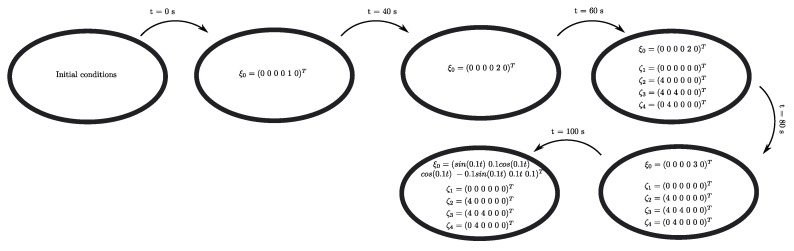
Simulation results—simulation flowchart.

The numerical simulation results of the second scenario addresses five arrangements integrated by four agents for 300 s. The complete maneuver in the 3D-space is depicted in Figure 8. As with the simulation for Scenario 1, the UAVs start from the initial condition depicted in Table 1. Then, the leader-following consensus protocol (27) is executed, being $\xi_{0}={\left(0\;0\;0\;0\;1\;0\right)}^{T}$ the virtual leader position, i.e., the reference point for the followers. Once the consensus has been obtained at the fixed reference point $\xi_{0}$ (which is achieved at t = 40 s), the virtual leader switches to $\xi_{0}={\left(0\;0\;0\;0\;2\;0\right)}^{T}$ and the consensus is achieved at t=60 s. Then, the control switches, and the leader-following formation protocol (46) is performed using an associate target location set $F=\{\zeta_{1},\zeta_{2},\zeta_{3},\zeta_{4}\}$, as the formation specification, given by

(47) $$F=\left\{\zeta_{1}=\left(\begin{array}{c} 0\\ 0\\ 0\\ 0\\ 0\\ 0 \end{array}\right),\;\;\zeta_{2}=\left(\begin{array}{c} 4\\ 0\\ 0\\ 0\\ 0\\ 0 \end{array}\right),\;\;\zeta_{3}=\left(\begin{array}{c} 4\\ 0\\ 4\\ 0\\ 0\\ 0 \end{array}\right),\;\;\zeta_{4}=\left(\begin{array}{c} 0\\ 0\\ 4\\ 0\\ 0\\ 0 \end{array}\right)\right\}$$

with $∥\zeta_{i}-\zeta_{j}∥=\varrho_{ij}$, where $\varrho_{ij}=\varrho_{ji}=4$ are the desired interagents distances. Note that i,j are such that (i,j)∈E and (j,i)∈E for the unidirected graph G={V,E} depicted in Figure 4. As before, only VTOL 1 receives information from the virtual leader. Then, for t>60 s and after a sufficiently long time, $∥\xi_{i}-\xi_{j}∥$ converges to $\varrho_{ij}=4$ and the formation shape is achieved at t=80 s. Once the formation shape is obtained by the four UAVs, the virtual leader state $\xi_{0}$ switches again such that the agents achieve an altitude equal to 3 meters ($\xi_{0}={\left(0\;0\;0\;0\;3\;0\right)}^{T}$) which is performed at t=80 s. After that, the $\xi_{0}$ is parameterized in time, that is $\xi_{0}={\left(\sin\left(0.1t\right)\;0.1\cos\left(0.1t\right)\;\cos\left(0.1t\right)\;-0.1\sin\left(0.1t\right)\;0.01t\;0.1\right)}^{T}$ for t>100 s to obtain the way points to follow for the UAVs and maintaining the desired shape. Figure 9 shows the five main phases during the simulation.

**Figure 10:**
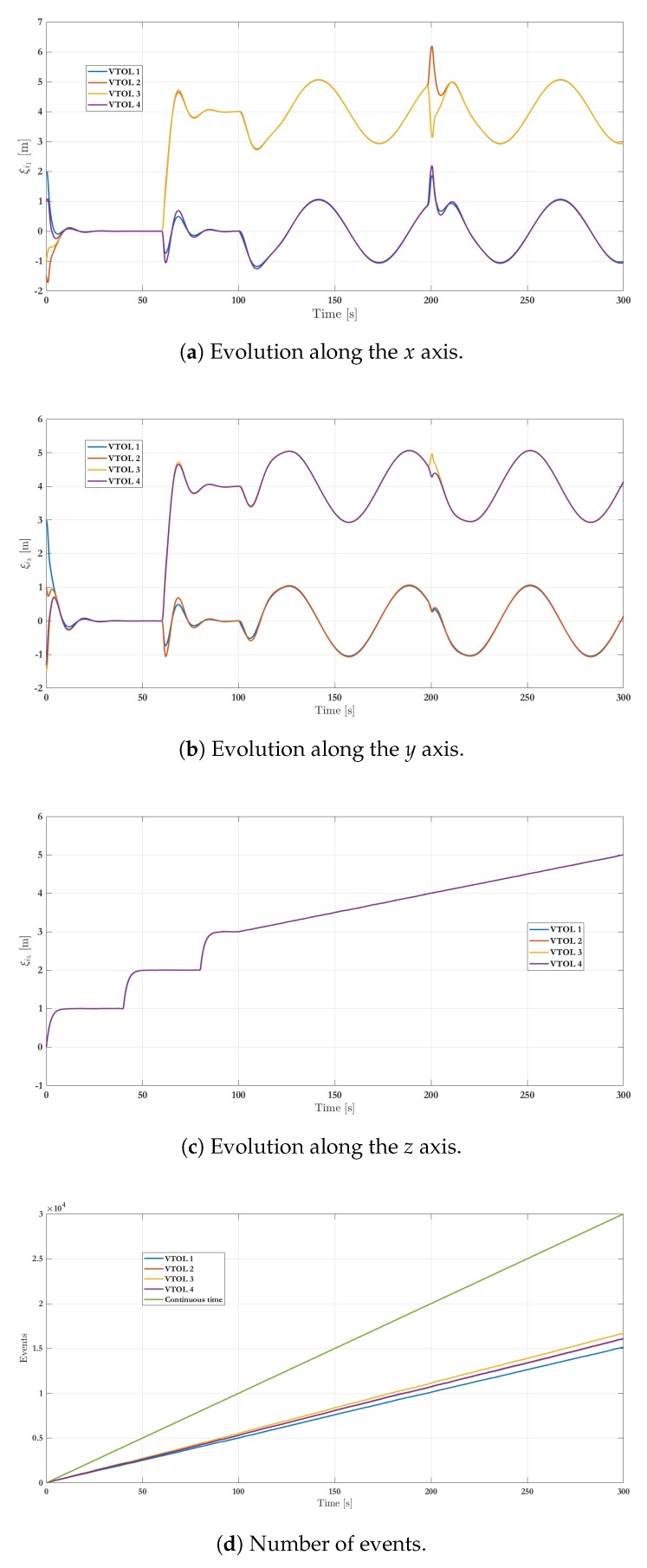
Simulation results—stage two: VTOL-UAV positions.

Figure 10a–c show the positions’ evolution of each agent for the second scenario. The simulation also sets an external perturbation on VTOL 1 at 200 s. Figure 10d exhibits the number of events for all agents. The plot also shows a continuous-time transmission among agents. Considering a sampling rate of 0.01 s during transmission and a simulation time of 300 s, there is a total of 30,000 events for each agent in the periodic case. In comparison, the event-based control strategy generates only 15,310 events for VTOL 1, 16,180 for VTOL 2, 16,780 for VTOL 3 and finally 16,210 for VTOL 4. This represents a reduction of about 51.03%, 53.93%, 55.93%, and 54.03% respectively in the transmissions. A video of this scenario can be viewed in the following link: Video of Scenario 2 (https://www.dropbox.com/s/jfuh058b5v33njb/Formacion_MDPI_2019.mp4?dl=0).

### 5.2. Experimental Results

**Figure 11:**
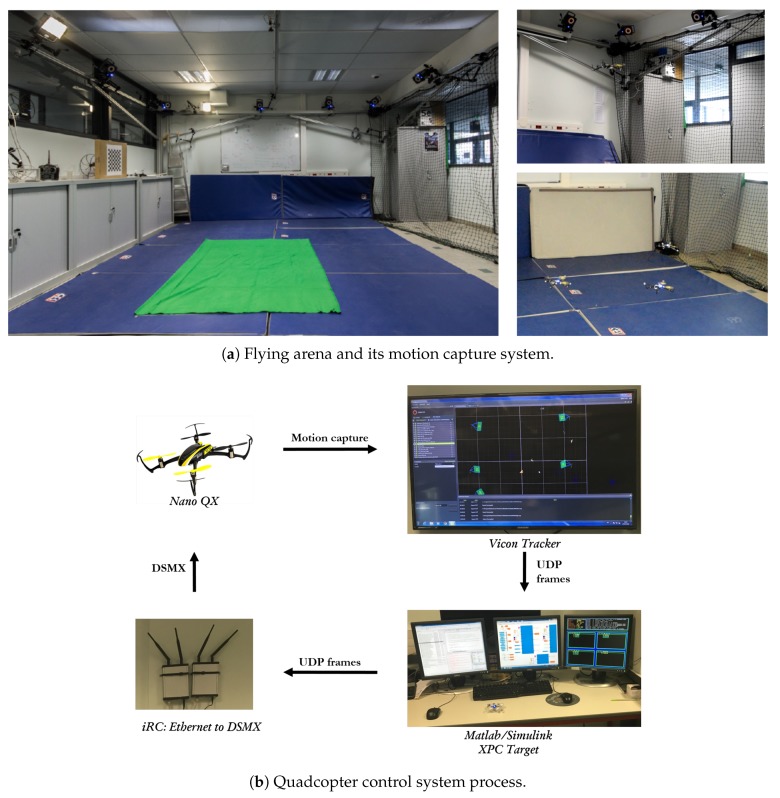
*MOCA flying arena* at GIPSA-lab laboratory.

The proposed control strategy has been verified in practice through a set of experiments. The selected vehicles are *Nano QX* quadcopters, whose on-board computer were modified to execute the control strategies. The attitude control for the quadcopter is implemented in an embedded system, which holds rate gyros and accelerometers for attitude estimation. Next, a ground station receives the position and estimates the velocity of the quadcopter utilizing a *Vicon Tracker* system and twelve cameras in the so-called *MOCA flying arena* at GIPSA-lab laboratory (see Figure 11a). The control strategy is implemented in real-time at 200 Hz on a computer using *xPC target* toolbox. The control signal is sent back to each quadcopter through a GIPSA-lab’s built-in bridge that converts UDP frames to Bluetooth or DSM2 protocol (see Figure 11b).

**Figure 12:**
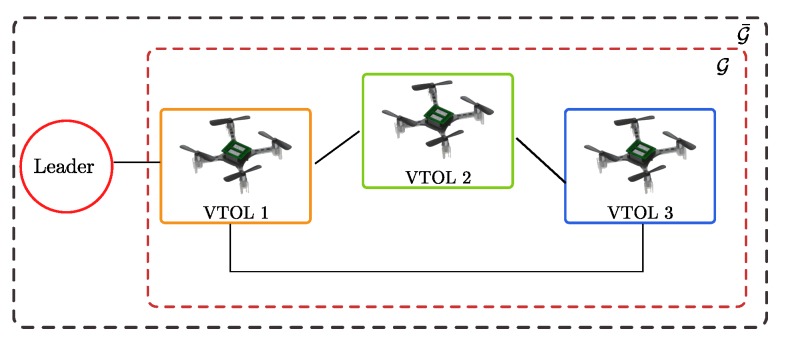
Graph for experimental results.

A collection of experiments are conducted to assess the performance of the proposed consensus and formation control. The tuning parameters and saturation for control law (11) are chosen to match with the actuators and vehicle characteristics. For the experiment, three agents are employed, each one being a nano quadcopter. The communication topology in the collaborative system is implemented via the graph illustrated in Figure 12, where VTOL 1 is the only agent that receives information from the virtual leader. The real-time implementation is performed for 120 s with a sampling time of 0.01 s.

In this experiment, the leader-following formation protocol (46) is executed and aims to ensure that the three vehicles keep two desired formation shapes described by the associate target location set $F_{1}$ and $F_{2}$, given by:

(48) $$F_{1}=\left\{\zeta_{1}=\left(\begin{array}{c} 0.5\\ 0\\ 0\\ 0\\ 0\\ 0 \end{array}\right),\;\;\zeta_{2}=\left(\begin{array}{c} -0.5\\ 0\\ 0\\ 0\\ 0\\ 0 \end{array}\right),\;\;\zeta_{3}=\left(\begin{array}{c} 0\\ 0\\ -0.5\\ 0\\ 0\\ 0 \end{array}\right)\right\}$$

(49) $$F_{2}=\left\{\zeta_{1}=\left(\begin{array}{c} 0\\ 0\\ 0.5\\ 0\\ 0\\ 0 \end{array}\right),\;\;\zeta_{2}=\left(\begin{array}{c} 0\\ 0\\ -0.5\\ 0\\ 0\\ 0 \end{array}\right),\;\;\zeta_{3}=\left(\begin{array}{c} 0.5\\ 0\\ 0\\ 0\\ 0\\ 0 \end{array}\right)\right\}$$

The desired height is governed by the virtual leader’s state which is set to $\xi_{0}={\left(0\;0\;0\;0\;1\;0\right)}^{T}$. For each vehicle, the initial conditions are depicted in Table 2 with the information of the attitude and position of each VTOL-UAV, where initial angular and linear velocities are zero.

**Figure 13:**
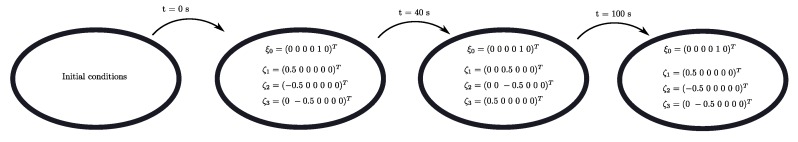
Experimental results: Flowchart.

**Figure 14:**
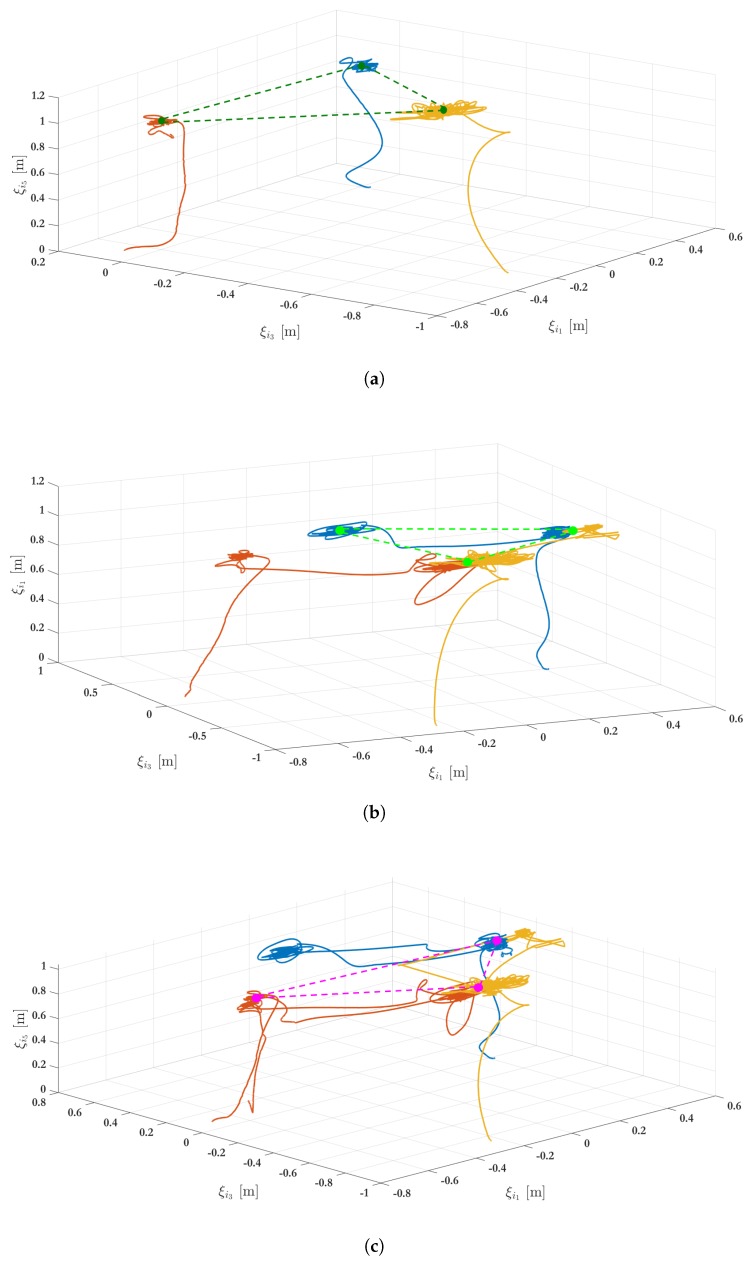
Experimental results: 3D representation. (**a**) Formation shapes described by the associate target location set $F_{1}$ and achieved at t=43 s; (**b**) Formation shapes described by the associate target location set $F_{2}$ and achieved at t=100 s; (**c**) One more time, formation shapes described by the associate target location set $F_{1}$ and achieved at t=180 s.

**Figure 15:**
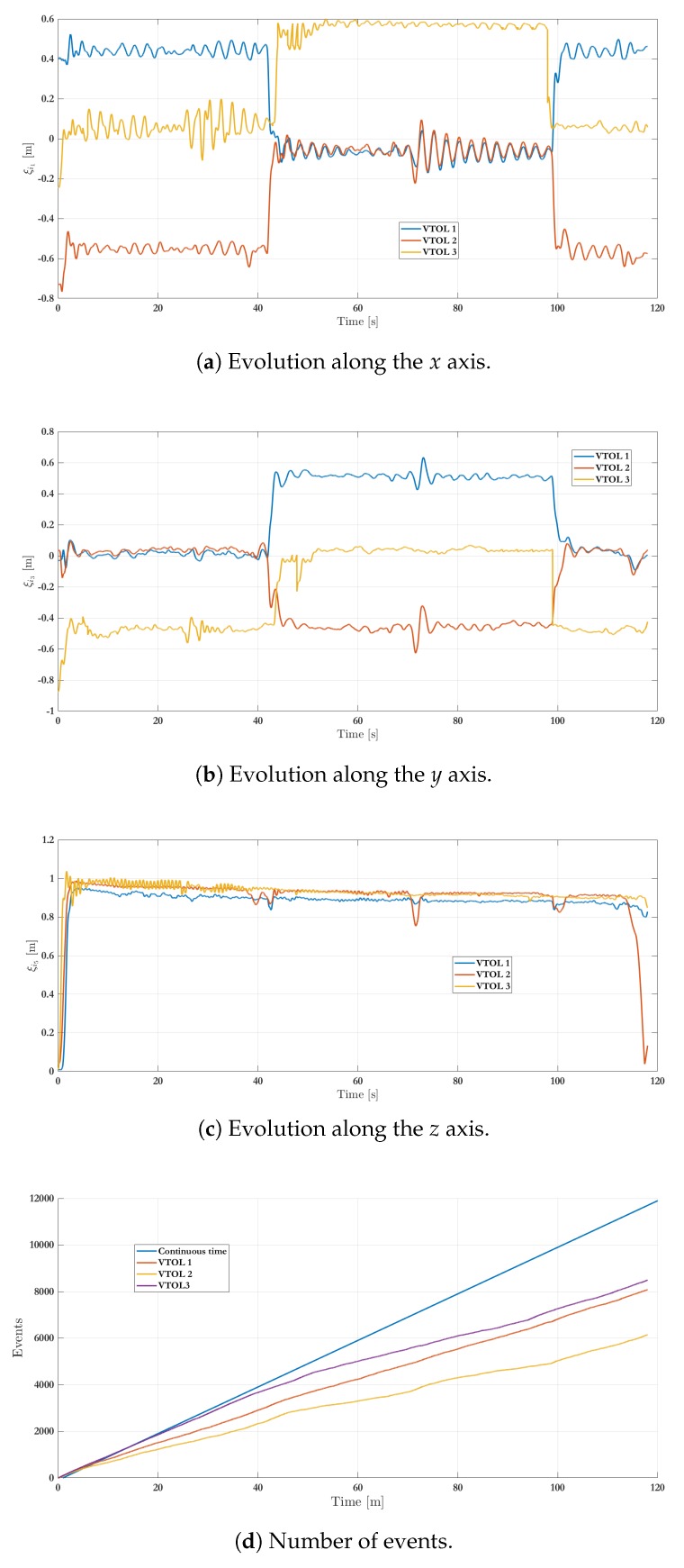
Experimental results: VTOL-UAV positions.

Figure 13 shows the three main phases during experimentation and the complete maneuver in the 3D-space is depicted in Figure 14. It is important to remark that the experimental setup considers every agent with given initial conditions to achieve the first arrangement formation given by $F_{1}$. Then, after 40 s, the arrangement is changed to the second desired position and is maintained until 100 s. Finally the collaborative system returns to the first arrangement. Figure 15 shows the position evolution, i.e., $\xi_{1}$, $\xi_{3}$ and $\xi_{5}$. The amount of events is also depicted in Figure 15d. Note that when an event occurs then the *i*th agent broadcasts its position and velocity to its neighbors. With the standard (periodic) frame and using a sampling time equal to 0.01 s, the state should be broadcasted 12,000 times by each agent within a span of 120 s. Using the proposed strategy, the number of events per agent is 8084 (VTOL 1), 6150 (VTOL 2) and 8493 (VTOL 3), which represent a reduction of the communication bandwidth utilization in the network of about 32%, 48%, and 29%, respectively.

## 6. Conclusions

This work presented an event-triggered distributive control strategy to give a solution to the problem of consensus and formation of a collection of VTOL-UAVs. The control strategy was designed, evaluated in simulation, and verified experimentally. Furthermore, a stability analysis of the whole system was provided: the event-triggered control guarantees trajectories with flexible limits of each vehicle and ensures practical convergence to the consensus or the formation. Due to the under-actuated nature of the VTOL-UAV vehicles, the inner–outer loop control approach was exploited. The inner-control loop is quaternion-based, and it is responsible for attitude and position stabilization, whereas the outer control loop is itself the agreement protocol, and is event-triggered. The maximal actuator capabilities for each quadcopter was also considered. Numerical simulations and practical experiments showed the performance of the proposed control strategy. The real-time implementation was performed using three nano VTOL-UAVs, and the information among them is carried out by a communication topology represented by an undirected and connected graph. Both simulation and experimental results highlighted that the proposed event-based control strategy correctly drives the collaborative system to the desired formations while highly reducing the communication resources, and consequently the energy consumption.

## Figures and Tables

**Table 1:** Initial conditions for the simulation results.

Agent	Attitude (ϕ,θ,ψ)	Position $\left(\xi_{i_{1}},\xi_{i_{3}},\xi_{i_{5}}\right)$
VTOL 1	(2, 8, −5)	(2, 3, 0)
VTOL 2	(10, −15, 4)	(−1.5, 1, 0)
VTOL 3	(−5, 10, −8)	(−1, −1.5, 0)
VTOL 4	(−15, 7, −2)	(1, −1.3, 0)

**Table 2:** Initial conditions for experimental results.

Agent	Attitude	Position
VTOL 1	( −3.1, 3.4, 0.8)	( 0.7, −0.6, 0)
VTOL 2	( 0.95, 0.99, 50.5)	( 0.4, −0.03, 0)
VTOL 3	( −0.9, 0.53, 2.72)	( −0.7, 0.03, 0)
